# Rethinking the Epidemiogenic Power of Modern Western Societies

**DOI:** 10.3389/fsoc.2021.638777

**Published:** 2021-06-04

**Authors:** Annabelle Lever, Lou Safra

**Affiliations:** Sciences Po–CEVIPOF, CNRS UMR, Paris, France

**Keywords:** COVID-19, industrialized societies, epidemic, public health, non-pharmacological measures

## Introduction

In less than a year, COVID-19 has imposed itself as one of the major health crises in the history of Western democracies although, as zoonotic diseases go, it is not especially deadly, nor are its symptoms especially terrifying (Harvard Health, 2020; Ioannidis, 2020). Faced with the spread of COVID-19, one of the most noticeable reactions of governments have been to impose lock-downs and important restrictions on freedom of movement and of association. These radical decisions caused such severe negative externalities (unemployment, poverty, inequality, loneliness, anxiety, depression, violence and the loss of schooling for millions of children) that they can only be temporary (Altman, 2020; Arpino et al., 2020; Banks, Karjalainen, and Propper 2020; Benke et al., 2020; Bonaccorsi et al., 2020; Czymara 2020; Green 2020; Grover et al., 2020; Martin et al., 2020; Usher et al., 2020; Fountoulakis et al., 2021). Such unparalleled restraints in peace-time were explicitly conceived as temporary measures to control, or mitigate, the spread of the epidemic while waiting for an effective and safe vaccine to be developed (See for instance Stella Kyriakides, EU commissioner for health and food safety, declaration on April 2020 Day, 2020). Even softer measures, such as social distancing and mask-wearing, were seen as ancillary to the main weapon—vaccination—and the assumption was that they had no relevance to healthcare in “normal” times.

Thanks to an unpreceded pharmacological effort, the development and roll-out of a variety of safe, effective vaccines against COVID 19 has occurred, offering concrete means to end this crisis (Zimmer et al., 2020). Nonetheless, these pharmacological solutions should not prevent the consideration of those factors, endogenous to our societies, which helped to catalize this pandemic. Indeed, this paper argues, epidemics reflect a combination of exogenous and endogenous factors. Attention only to the former risks underestimating what we call 'the epidemicogenic' dimensions of our society, at the risk of leaving us yet more vulnerable to destructive epidemics in future.

## Issues With Pharmacological Solutions

The European Commission and the World Health Organization have both emphasized the importance of ensuring that pharmacological tools against Covid-19 are developed collaboratively and made accessible to all equally, in order to counter the medical, political and social risks of the pandemic (Riordan, 2020; World Health Organization 2020). They fear, rightly, that otherwise scientific developments on which everyone depends will be instrumentalized by those who are able to monopolise them, and used to advance sectarian political, economic or ideological agendas. Within countries, not merely internationally, differential access to healthcare can be used to favor one social groups over others, and to discriminate on racial, religious and ethnic grounds (as it has already been the case for food distribution (The New Humanitarian, 2002) and the international context makes such problems possible, if not probable. In July, already, Putin announced the development of a safe vaccine against COVID-19 by Russia (Official website vaccine against coronavirus Sputnik V, 2020). This announcement places Russia as a leader in technological development with all its potential consequences, including the military ones. The name of the vaccine itself, Sputnik V, reveals the reputational, diplomatic and political intentions behind the announcement.

Besides these political and ethical considerations, the long-term consequences of relying on pharmacological solutions for epidemics are not promising; scientists have indicated that pandemics may be more and more frequent in the future, due to the erosion of natural habitats exacerbated by global warming, agricultural intensification and urban areas extension (Dasgupta and Andersen 2020; Epstein, 2000; International, 2020; Madhav et al., 2017). Specifically, by causing an average temperature rise and increasing contact between humans, livestock and wildlife, these practices create the ideal conditions for the development and spread of zoonotic diseases. However, vaccines, in their nature, are disease-specific, and thus an inefficient way of protecting global public health. Successful vaccines for global health threats of any severity and scale require the dedication of colossal disease-specific resources, as we have seen, with the real possibility of extensive restrictions on civil liberty in the meantime. Considerable emphasis has been put on the economic and mental health consequences of the COVID-19 pandemic, but little attention until now has been paid to the consequences of mass deaths for families and the generational balance among populations, although as know from the HIV epidemic in Africa, these can be severe. Moreover, the psychological effects of acute exposure to the risk of infectious disease have significant political and social consequences in themselves. Epidemics tend to produce xenophobic scapegoating and hostility to ‘outsiders’ (Clissold et al., 2020; Schaller, 2011; White, 2020)—in this case, Asian communities in the West have borne the brunt of that hostility since Spring 2020. Such empirically observable tendencies appear also in experimental psychology research, which shows that real or perceived exposure to a threatening pathogen increases racist and xenophobic attitudes in test participants (Aarøe et al, 2017; Faulkner et al., 2004; Millar, Fink-Armold, and Lovitt 2020). So instead of looking for made to measure, post-hoc, solutions to each large-scale epidemic, it is crucial to find ways to prevent them and, where that is impossible, to minimize their severity.

## Diseases as External Enemies: The Exogenous Dialectic

Mainstream political discourse presents epidemics as an external enemy and epidemic prevention as a process of repelling this external threat. Emmanuel Macron’s reference to wartime, in his speech announcing the France’s lockdown in March 2020 exemplified the phenomenon (Macron, 2020). The representation of epidemics as something external fits naturally with intuitive Western images of disease as an alien threat to the body. Pathogens worm their way into the population and cause an epidemic in just the same way that they insinuate themselves into individual bodies and cause disease (Napier, 2020). Although widely shared in Western societies, this view is far from universal. For example, the Balinese view of immunity is based more on the idea that humans play an active role in the circulation of viruses than on the idea that they are external threats to the self (Napier, 2020). Importantly, the exogenous representation adopted by Westerners faces two problems. First, it ignores the fact that acute infections often give rise to chronic illness and, even when cured, can damage one’s ability to fight further infections in future some illnesses, can leave one vulnerable to new infections over the long term, as well as to the chronic illness over a life time which acute infections often entail, (e.g. Bonaccorsi et al., 2020; Musher, Abers, and Corrales-Medina 2019; Sejvar, 2007; Xiong et al., 2020). In the case of COVID-19, specifically, an increasing number of incapacitating long-term pathologies have been noted by the medical community, and it is quite possible that the full range of long-term damage from COVID 19 has not yet been appreciated (Carfì, Bernabei, and Bermpohl; CDC 2020; Fraser, 2020). However, the second main problem with the idea that disease, and particularly epidemics, can be adequately conceptualized as an external threat to an otherwise healthy body/society is that this ignores the social conditions that make epidemics possible, and which help to determine who will suffer most from them.

The exponential spread of COVID-19 in Western countries, therefore, forces us to consider the causal role of our structuring institutions, habits and norms in turning what might have been a relatively contained disease into an epidemic that had hit us full force by March 2020. Indeed, in Autumn as in Spring 2020, most Western countries experienced exponential rises in the number of COVID-19 cases (Roser et al., 2020). Thus even if this particular epidemic emerged from China, and was made worse by the persecution of journalists and doctors there, (e.g. Waterson, 2021), Western societies have clearly played a role in its propagation and development–in part, through their reluctance to accept that the epidemic could be controlled without access to a vaccine, as seems largely to have happened in South East Asia (An and Tang 2020). As Ludovic et al. (2020) have highlighted, integration into a globalized market economy has been one of the drivers of the COVID-19 pandemic. Indeed, countries with higher levels of trade also experienced a higher number of COVID-19 cases in april 2020. While the increased circulation of people between countries is undoubtedly a factor in this association, the question arises as to whether this effect has not also been mediated by the very structure of modern Western societies.

## The Structure of Western Modern Societies as Epidemic Accelerators

Significant outbreaks of Covid-19 in slaughterhouses throughout Europe (Académie nationale de ) have reminded us of the importance of healthy working and living conditions in containing lethal epidemics, such as *tuberculosis*, diptheria, and measles long before there was a vaccine for them, or, as in the case of cholera, in controlling diseases for which there is no vaccine (Porter, 2005). Slaughterhouses are poorly ventilated and crowded workplaces and their employees often share cramped, unsanitary housing conditions as well. Whereas the importance of adequate ventilation has only recently been stressed as an essential tool in controlling the spread of COVID-19 (Connolly, 2020), its importance to the control of *tuberculosis* was known since the late 19th century (Dubos and Dubos, 1987). More recently, the importance of fresh air and sanitary conditions to the health of non-human animals was highlighted by the role of intensive livestock farming in the spread of zoonotic diseases. Intensive poultry farms suffer more from the development of coccidiosis, a potential fatal disease for chickens, due to the over-crowding of animals (Tewari and Maharana 2011), and “mad cow disease’”—less colloquially known as Bovine Spongiform Encephalopathy—is thought to have arisen as a result of feeding intensively farmed cattle with meat-and-bone- meal (Colchester and Colchester 2005). Its human form, Creutzfeldt-Jakob disease is thought to have been transmitted by eating contaminated cattle. In short, the importance of fresh air, low density housing, safe drinking water, food safety and effective waste collection were all key to the control of epidemics before the advent of modern treatments for them. However, it seems that repeated zoonotic epidemics are necessary to remind us of their continuing relevance nowadays.

A critical look at the organization of many industrialized societies highlights the contrast between classic public health measures, and current ways of living in our societies, with their high population density, constant population movements and life, work and travel in low ventilation indoor areas. Even in hospitals and doctors’ offices fresh air can be hard to come by, and in the United States, notoriously, one is not supposed to open windows because centralized heating and air conditioning mean that this otherwise innocent gesture risks over-heating or freezing hapless co-workers elsewhere in the building.

Moreover, the social organization of contemporary industrialized societies appears at odds with the primary principles of public health. While “case isolation” has been one of the central elements of the strategy of the World Health Organization for managing COVID-19 epidemic, this principle cannot be implemented given the current constraints on workplace organization. In addition to the statutory and economic constraints on sick leave, such as the existence of unpaid days during paid sick leave, Western societies, create a moral and reputational incentive to work, even when feeling unwell, because of the importance that they attach to being “productive.” Moreover, health insurance for cattle and crops, as well as for people, are generally available only at significant personal cost, although if widely used they would promote an important public good: the rapid sharing of reliable information about infections before they become epidemics. As such, industrialized societies unite the material and psychological characteristics that spread disease. This social organization is at odds with the natural behavior individuals adopt when threatened with disease. Psychological studies in experimental settings show that individuals tend to avoid contact with potentially infectious individuals (Schaller and Park 2011; Park, Van Leeuwen, and Chochorelou 2013; Blacker and LoBue 2016). Such individually and collectively beneficial behavior, unfortunately, is hard to adopt in societies where people are forced to work in close contact with others, and to commute using overcrowded public transport.

## Understanding the Roots of the Epidemiogenic Power of Western Societies

However, if the factors that contribute to epidemics are well-known and, in a sense, part of folk knowledge, we can wonder why industrialized societies are designed the way they are. The rise of a productivity and growth-centered economy since the Second World War is an important factor. Two other factors also strike us as essential to current vulnerabilities to epidemics, though so far, they have not received attention. The first, is the compartimentalization of society, such that problems of human health are largely separated from the health of non-human animals, pollution and the design of our physical and social environment. Not only does this make it difficult to recognize changes in public health which are relevant to epidemics, as in the case of pollution (Fattorini and Regoli 2020; Frontera et al., 2020; Travaglio et al., 2021), but it makes it harder to integrate knowledge across public policy areas.

Second, with the successful development of vaccines and antibiotics since the Second World War, diseases such as diptheria, measles, *tuberculosis*, polio have become distant threats for many of us, rather than omnipresent threats to families and, especially, to their most vulnerable members. vulnerable members. Although the 21st century is not exempt from life-threatening transmissible diseases, in most industrialized countries these mainly concern specific “at-risk” populations rather than everyone: older people for the seasonal flu, LGBT communities and drug addicts for AIDS, prisoners and deprived urban populations for *tuberculosis*. In other words, mass infectious diseases have become less and less of a concern for public policies in most countries, and outbreaks are publicly treated as isolated and exceptional events. In this context, public health principles are increasingly ignored in the development of contemporary societies, paving the path for the exponential spread of epidemics.

## Conclusion

In this paper, we highlight the structural, epidemiogenic factors in industrialized societies that contribute to the development, intensification and spread of epidemics. While these factors are generally acknowledged when dealing with epidemics in non-human species, their significance for humans was only slowly recognized in the early stages of the COVID-19 pandemic. Reconsidering the structural factors that place our societies on a collision course with the most basic lessons of public health is the only way to design long-term solutions to current epidemics, and to prevent further ones in future. Recognizing and changing those factors is, also, the only way to combat the socially inegalitarian patterns of risk and illness, which COVID-19 has laid bare (Wolff and de-Shalit, 2007; Wilkinson and Kate, 2009). Crowded, unsanitary housing; poorly ventilated and dilapidated schools; poorly paid work, with no entitlement to sick leave or parental leave, means that the burdens of COVID-19 fall hardest on socially vulnerable groups who, too often, are the offspring of parents and grandparents who, themselves, suffered discrimination, exploitation and neglect (Patel et al., 2020). In short, recognizing and correcting the endogenous determinants of epidemics is not simply a matter of prudence, or of wise public policy. It is also a matter of justice, and of our duty for future generations.
